# Tradition and reinvention: the making and unmaking of herbal medicines in the UK

**DOI:** 10.1111/jols.12367

**Published:** 2022-05-22

**Authors:** NAYELI URQUIZA‐HAAS, EMILIE CLOATRE

**Affiliations:** ^1^ Law School University of Kent, Giles Lane, Eliot College Canterbury CT2 7NZ England

## Abstract

This article looks at the development of the regulation of traditional herbal medicines in the European Union (EU) context and its effects in the United Kingdom (UK). Drawing on socio‐legal encounters with science and technology studies (STS), it explores how UK and EU stakeholders have struggled to regulate herbal products, and suggests that in order to tackle growing concerns about their safety, emerging EU legislation built on socio‐technical imaginaries of ‘tradition’. We argue that in doing so, the law also reshaped herbal medicines in the UK, rewriting their histories and potential futures by fostering new practices of herbal medicine making that sit precariously on the boundaries of what is lawful. Through an empirical exploration of the everyday landscape of herbal medicine in the UK, this article shows how the label of ‘tradition’ embedded in the new legislation transformed and unsettled the existing material practices and relationships that had underpinned herbal and traditional medicine.

## 1 INTRODUCTION

In 2004, the European Union (EU) adopted the Traditional Herbal Medicinal Products Directive. This Directive set up a complex new regime of authorization for herbal medicinal products that fed into and intensified ongoing debates about herbal medicine in the United Kingdom (UK). In time, it triggered a significant shift in the landscape of herbalist practice, creating new fault‐lines to how practitioners make herbal medicines. In this article, we review the history of the Directive, and of the shifts that ensued, paying particular attention to the Directive's explicit focus on ‘traditional’ herbal products. One of the most contested and politicized concepts in debates around healing, ‘tradition’ had a significant and impactful framing. The Directive translated the term in a rather technical way, predicating the legitimacy and legality of traditional herbal products on their standardization and industrial transformation. In doing so, it fitted herbal medicines into a particular vision of what contemporary (and future) healing should look like. Yet it obscured competing claims of what constitutes traditional herbal medicine, and of how it could contribute to healthy futures. In this article, we analyse these processes through the notion of ‘imaginaries’, exploring how competing world‐making visions underpinned debates around traditional herbal medicine and the characteristics of those that became embedded in the Directive. Highlighting the particularity of the Directive's definition of ‘tradition’, and its relationship to the history of herbal medicines in the UK, we then turn to its effects in practice, observing how those who disagree with this (re)definition of their traditions have sought to resist or evade the impact of the law. While focusing on a particular debate and case study, the article also raises themes of relevance to broader socio‐legal debates. In particular, it interrogates the role of the law in socio‐technical world‐making projects and explores the everyday responses of those who want to pursue alternative futures, while reasserting a different kind of past.

Though medicinal plants have been used in healthcare practices for centuries, their regulation has proven controversial. Perceived as both powerful and potentially dangerous, especially when used in the wrong way or without adequate knowledge, they have long been seen as needing some kind of oversight. However, plants are ubiquitous; controlling who uses them and how is not always feasible, and states have been well aware of their own limitations. Herbal medicines can also take many forms, from their natural raw state to highly transformed materials produced industrially, and can be marketed for health, well‐being, or cosmetic use, or sold as food supplements. In addition, the use of plants is often shaped by culture, beliefs, or individual and collective traditions, and built around human relationships (such as with healers, stores, or brands that inspire trust and confidence) that can matter almost as much as the plants themselves. As a result of these challenges, the regulation of plants in the UK has historically been shaped by compromises and exemptions. Even when the state tightened the regulation of medicines, it also opened up a space for herbalist practice to be maintained and medicinal plants to be used without being subject to the heavier checks imposed on pharmaceuticals. Home‐grown practices of plant‐based health care and more recently imported ones (such as Ayurveda or traditional Chinese medicine) thrived under the legal exemptions, embedding themselves in the day‐to‐day lives of numerous users and practitioners.

The exemptions from tighter regulation granted to medicinal plants has been a cause for concern for some parties throughout history. However, regulatory anxiety increased from the 1980s, intensifying in the following decade. At that time, the markets for medicinal plants were undergoing significant changes; more industrial products were circulating, in the UK and beyond, embedded in global markets with often limited traceability, and accessible more directly to consumers through shops, and later online distribution. While some of these products were integrated into pre‐existing herbalist practices, others produced very different types of self‐care practices as well as over‐the‐counter remedies.

Throughout the 1990s, it became clear that some of these products could have significant harmful effects on users’ health, due to poor quality, adulteration, cross‐contamination, or ill‐advised usage. Soon, safety concerns led to calls for tighter regulation of herbal products; previous accommodations for those ‘not‐quite‐medicinal’ products were judged inadequate in the face of new health practices and markets. In the UK, this call for regulation became entangled in political tensions around the need to preserve a less heavily regulated space for natural health care and to protect the cultural practices that underpinned the use of some herbal medicines. Even though the global markets for new‐generation pills and capsules may be quite different from the practices of many herbalists, the two spheres seemed inseparable, and parliamentary attempts to regulate the former were seen as a threat to the latter. In parallel to UK debates, similar concerns emerged at the EU level, where the growing market for (industrialized) herbals was seen as creating both safety threats and complications around cross‐border trade due to the lack of uniform regulation. The UK government, faced with deadlock in national legislation, turned to the EU as a forum to regulate the safety of herbal products, playing a key role in what would become the EU's Traditional Herbal Medicinal Products Directive.

The Directive effectively created a regime for some herbal medicines to be allowed onto the market through less stringent regulations than those imposed on pharmaceuticals. However, it also had broader ramifications; rather than simply regulating ‘traditional herbal medicines’, the Directive came to redefine the boundaries of what should be considered as ‘traditional’ products, and the conditions under which they should be allowed to enter markets. In doing so, it shaped the future of ‘traditional’ herbals as one that would look determinedly more industrial, and arguably distanced from other ideas of ‘tradition’. Even looking only at the UK, the effects of the Directive on those who do not share its understanding of ‘tradition’ are notable; pushed further to the fringes of healthcare practice, they have had to negotiate with newly proposed legal versions of what ‘tradition’ means for herbal medicines, and which aspects of it deserve to be free from regulation.

In this article, we reflect on these developments, focusing on how the notion of ‘tradition’ has been transformed throughout regulatory debates and processes. We suggest that the Directive produced new categories of herbal medicines and embedded particular visions of pasts and futures in the law. To capture the world‐making nature of legal processes, we rely on the science and technology studies (STS) concept of ‘imaginaries’. This concept allows us to look specifically at how the process of law making involved negotiating between different visions of the pasts, presents, and futures of herbal medicine as a healthcare technology, and created boundaries of acceptability, along with new definitions of ‘tradition’ and their suitability to contemporary health care. Focusing on the UK as a case study, we interrogate how these law‐making (and world‐making) processes impacted the everyday practices of those whose understanding of ‘tradition’ in herbal medicine differs from that of the law.

## 2 HERBAL MEDICINES IN THE UK

Herbal medicine has a long history in the UK. Locally, it finds its roots in folk healing practices transmitted orally, some of which were revived by social and professional movements across the nineteenth and twentieth centuries.^1^ It has also experienced intense cross‐fertilization; knowledges, people, and materials have travelled throughout the British Empire, and later all around the world with the expansion of global markets.^2^ As is the case with other healing practices, migrants have often brought their medical traditions to the UK, creating new hybrid practices.^3^ Herbal medicines have also crossed paths with a multiplicity of healing practices and cultural movements. Overall, medicinal plants are embedded in individual histories that have transformed their meanings and their use over the years.

Herbal medicine also rests on a wide spectrum of products, materials, and processes. For example, some practitioners and users rely primarily on simple decoctions or teas, using unprocessed plants as their main material. Others rely on more transformed products, with plants being used as the source of active substances for elixirs, syrups, extracts, and powders. Since the 1990s, a growing market for ‘natural products’ has also offered users new types of pills, drops, creams, and capsules that, though based on medicinal plants, look much more like pharmaceuticals. The kind of networks that are involved in the making of herbal materials similarly vary greatly. Some networks are fairly simple, sustained by so‐called ‘cottage industries’, which distribute raw materials or basic manufactured preparations requested by herbalists; others involve more intermediaries that sometimes double up as providers of either raw plants or ready‐made pills manufactured by larger‐scale producers. While some of the networks are local or national, many cross borders, feeding into a significant global market for medicinal plants and products. Practices that have originated beyond the UK (such as traditional Chinese medicine or Ayurveda) are of course more likely to rely on such transnational networks and medicines grown or produced elsewhere.^4^ As well as networks of production, herbal medicines depend on a variety of networks of care. Some products are available in shops, supermarkets, or online, and can be bought and used by patients with no intervention from healers; however, professional herbalists also occupy a significant place in health practices in the UK, advising users, supplying products, and often assembling bespoke formulas in their own dispensaries. Unlike pharmaceutical drugs, which tend to have only one active substance, herbal and traditional medicines have complex mixtures, and actors along the chain, from herbalists to suppliers, have developed expertise in assembling such mixtures in their many forms.^5^ In everyday practice, herbal medicine relies on several rhetorical registers, in which the words ‘natural’ and ‘tradition,’ for example, occupy a significant place. Regularly used in preference to biomedicine, which is regarded as ‘harsh’, herbal products are often perceived and presented as mobilizing both the gentleness of nature and its powers and as relying on knowledges transmitted over generations. This does not exclude the mobilization of scientific registers; science can be mobilized as a way to explain why a particular product or technique should be seen as safe, reliable, and/or effective. In this way, herbal medicines rely on several layers of legitimacy, mobilized and adjusted to fit the particular understandings of experts and providers or to appeal to users and consumers.

UK laws have created a relatively open space for herbal practice. When the 1968 Medicines Act significantly tightened the regulation of medicinal products, herbalists mobilized to ensure that these new rules would not have the effect of outlawing their practice. They eventually secured special provisions for some herbal medicines. Under Section 12(1), the law provided an exemption from licensing for any herbal medicine made at home or in a business area after a consultation. In addition, Section 12(2) exempted over‐the‐counter herbal medicines from licensing as long as they were prepared through non‐industrial methods (defined as crushing, drying, and comminution) and the labels on the product did not contain any medicinal claims.^6^ Over the years, practitioners developed a lay understanding of the law, tolerated by the state, that stretched Section 12(2) to mean that they could ask third parties to mix a remedy for a particular patient, or that they could mix their formula for generic conditions, such as a herbal skin remedy or cough syrup. The exemptions became gradually more difficult to justify. In particular, from the 1990s, they were seen as partly to blame for incidents linked to plant‐based products from the Ayurvedic and traditional Chinese medicine markets. Some products sold under the herbalist exemptions were shown to contain toxic substances, others were different from what they were claimed to be (for example, with toxic plants substituted for non‐toxic ones), and some mixtures contained pharmaceutical substances.^7^

This was happening at a time when the market for herbal medicines was also changing at a fast pace, with the influx of a new generation of plant‐based products reputed to have some health benefits circulating under different guises (for example, food supplements as well as herbal medicines). These products blurred the boundaries between ‘modern’ and ‘traditional’ in herbal medicine; while packaged and processed in ways that could appeal to customers seeking the reassurances of modern technoscience, they also emphasized that the plants that they used belonged to a long‐standing tradition of herbal medicine. Taken together, the frictions that appeared around risky products, and the fast‐changing nature of the market for herbal medicines, brought new pressure on the existence of the herbalist exemptions, generating new regulatory debates throughout the 1990s. In these debates, pressure to improve safety also mobilized and transformed the legal and technical framings of the concept of ‘tradition’.

## 3 ‘TRADITION’, IMAGINARIES, AND LEGALITIES

In the analysis that follows, we explore how the regulation of herbal medicines developed from the 1990s onwards, and how new legal framings came to transform everyday practices. We focus in particular on the ambiguity of the concept of ‘tradition’ in those debates, and what this implies about the interface between legal mappings and social relationships in healing. The use of the term ‘tradition’ in the context of the Directive is significant; ambivalent and mobilized to different effects by different actors, it is one of the most volatile terms in healing debates.

STS scholars and historians of medicine have long stressed how the word ‘tradition’ and associated rhetoric can be used both by healer communities and by political and legal actors to produce particular classifications that do not always map easily onto networks and practices but fulfil other purposes.^8^ For example, Tilley demonstrates that African countries in the 1960s mobilized the discourses and materials of traditional medicine as symbols for new techno‐scientific political possibilities, and as engines of economic growth.^9^ Rather than describing a given reality, the notion of ‘tradition’ is performative, producing identities and embedding distinctions and boundaries that shape rather than simply reflect lived experiences of healing. Instead of operating within defined spaces such as ‘modern’ or ‘traditional’ health care, healing practices borrow from a multiplicity of techniques, materials, and knowledges and adapt across space and time to new demands, new opportunities, and new ideas. Framing such hybrid practices as either ‘traditional’ or ‘modern’ can help to situate them in specific social movements or support claims to legitimacy. At the same time, doing so has material effects; for example, a product sold as a traditional medicine may be packaged in a way that appeals to a sense of the past, and healers’ codes and practices may play on registers that echo particular framings. The authorities play a key part in the deployment of narratives of tradition and modernity, including through the deployment of legal instruments. These draw boundaries, creating normative spaces for some healing practices and materials and excluding others. For example, as we will see in our case study, ‘tradition’ may be perceived as offering exemptions to particular medicines and practices.

In this article, we explore such mobilization of ‘tradition’ in the context of the EU and UK legal regulations of herbal medicines. We interrogate the negotiation of the concept of ‘tradition’ not as a standalone rhetorical tool but as part of a broader ‘imaginary’: an imagined future in which technological solutions would leave a negotiated space for acceptable remnants of the past. In doing so, we aim to locate the mobilization of ‘tradition’ at the crossroads of discursive and material practices, while seeking to capture the world‐making powers of law and the counter‐narrative offered by other agents.^10^ The concept of imaginaries upon which we draw is derived from STS, though its implications for legal scholarship have also been explored.^11^ Jasanoff and Kim define socio‐technical imaginaries as ‘collectively imagined forms of social life and social order reflected in the design and fulfilment of nation‐specific scientific and/or technological projects’.^12^ Originally, the concept was used to reveal how the governance of new technologies is rooted in particular state visions of national identities, imagined pasts, and desired futures. Often focusing on states’ attention to the promissory futures of emerging technologies, but also on how states perceive their role in dealing with risks or the place that they see science as occupying in their pasts or futures, STS work on socio‐technical imaginaries has demonstrated the world‐making powers of such visions. Without ever being fully self‐fulfilling, imaginaries perform particular realities, playing a part in determining the directions that governance and regulation take, and in turn, the possibilities left for particular technologies to emerge (and in what form).

At the crossroads between socio‐legal studies and STS, scholars have reflected on the role of legal regulations in producing or sustaining particular socio‐technical imaginaries – and indeed the role of socio‐technical imaginaries in producing or sustaining particular legal regulations. Legal texts and procedures play a central role at both levels. As expressions of state ambitions, they embed broader visions of science and technology;^13^ at the same time, they come to produce some of the key constraints that will be imposed on future possibilities. Legal texts, laws, reports, and speeches are important both because of the requirements and expectations that they set, as regulatory tools, and because of their role as discursive frames. In defining terms and setting out the conditions of legal existence of specific products, they delineate the material embodiment of particular realities and shape the form and boundaries of practices, experiences, and knowledge.^14^

Imaginaries are by nature disruptive of socio‐technical temporalities; they reshape the present by projecting imagined futures. This process is solidified when they are embedded in law, constraining future possibilities along the lines sketched at a particular moment in time, with a given understanding of socio‐technical futures, and layering and distorting temporalities.^15^ In our case study, an additional factor is that the future visions deployed are also about the past(s) of herbal medicine and how the law confronts questions around healthcare products. This means that regulating ‘traditional’ herbal medicines is also about facilitating, hindering, or conditioning the travels of past knowledges into future health practices. In this process, some past practices are deemed more acceptable than others, and some are rendered invisible. Here as elsewhere, imaginaries are always partial, produced by situated individuals or groups who hold their own politicized visions of acceptable futures. However, even where such visions become embedded in law, others continue to perform their world‐making projects.

This article looks at how the legal category of traditional herbal medicines emerged after state actors increasingly framed plants as risky objects that needed better regulation. As a frame, ‘tradition’ enabled some plants to be seen as safe enough to benefit from a light‐touch regime, but only if manufacturers abided by specific conditions; traditional herbal medicines were constrained in rather modern ways, with scientific and technological knowledge used to guard against their potential risks. In doing so, the Directive conjured a future in which the EU medicine governance would be steered through technoscience. This vision also drew on existing resources and political inclinations, particularly in its reliance on biomedical logics to resolve public health dilemmas.^16^ This projection of the future clashed with other understandings that were born out of the multiple realities that had shaped the use of and methods of making plant‐based medicines in the UK. As we will see, there were important social and political dimensions of herbalist practice generating other understandings of ‘tradition’ that were eventually squeezed out of the Directive and the corresponding regulations.

Maybe because of disciplinary boundaries, there has been limited consideration of how imaginaries that are embedded in law affect its impact in practice. Yet one of the effects of regulation is also to cement the shape of socio‐technical relationships to come. In the final part of this article, we interrogate what happens to those whose own aspirations do not align with the kind of future sketched by the law. Specifically, we explore how those who aspire to different herbal futures based on small‐scale relationships, rather than industrial standardization, have responded to the EU's attempt to redefine the meaning of ‘tradition’. In this part of the article, we build on socio‐legal scholarship on legal consciousness to interrogate the forms of everyday resistance deployed by UK herbalists, and how conflicting imaginaries influence legal relationships.^17^ Our case study focuses on actors on the fringes of the law, who negotiate with its letter while resisting its spirit, offering a particular type of ethical challenge to the world‐making attempts of regulators.^18^ In challenging both the technical requirements of the law and the underlying vision that it proposes of how herbal medicines can be legally accommodated in contemporary healing practices, herbalists offer their counter‐narratives of ‘tradition’ in herbal practice. They deploy micro‐tactics to continue to practise, operating on the borders of legality and illegality, visibility and invisibility, to sustain world‐making projects that the law has rendered more difficult. Those who maintain a certain faith in the authority of the law nevertheless acknowledge their alienation, both in terms of the visions that it embeds and in terms of the practicalities of sustaining the kind of practices that regulators have sidelined in their redefinition of ‘tradition’.

## 4 METHODS

Our analysis focuses on the period between 1980 and 2012. We examine how calls to tighten the regulation of herbal medicines started to emerge in the UK and the EU, culminating in the Human Medicines Regulations of 2012. It is based primarily on documentary analysis, supplemented by semi‐structured interviews and some observations. The documents that we use are legal and policy documents (parliamentary debates, written questions, and consultations) and some grey literature produced by herbalists and their associations. We also build (particularly in the final part of this article) on interviews with two managers at regulatory bodies in the UK, representatives of four herbalists’ associations (representing the main streams of herbalism in the UK: Western herbal medicine, traditional Chinese medicine, and Ayurveda), a representative of a herbal medicine manufacturers’ association, and three independent herbalists (three of the four representatives of associations interviewed were also practitioners and responded from a dual perspective). We also rely on material available in the public domain that reflects the views and experiences of herbalists (including podcast interviews with herbalists and debates between herbalists in online forums) and on ethnographic observation and note taking at public meetings and events around herbal medicine held in the UK between 2017 and 2019 (two academic events led by phytomedicine researchers, two meetings of herbal medicine practitioners, and two meetings organized by producers and manufacturers). This research is part of a larger project on the regulation of traditional and alternative therapies across selected European and African countries, which has also informed the questions raised in this case study, and our analysis.

## 5 NEGOTIATING HERBAL MEDICINE REGULATION

### 5.1 From tolerance to risk management: regulatory concerns in the UK

Concerns about the quality and safety of medicinal plants started emerging from the 1980s across the EU. They were particularly acute in the UK, where 80 per cent of herbal medicines in the market were unlicensed; given the influence of the UK on EU health regulation, this would have repercussions in debates to follow.^19^ Domestically, the regulation of herbal medicines proved to be a contentious political issue, with resistance to governmental strategies from both parliamentarians and associations who relied on plant‐based medicines.

The main impetus for regulatory change in the UK came from the Medicines Control Agency (MCA), now renamed the Medicines and Healthcare Products Regulatory Agency (MHRA). Throughout the 1990s, the MCA was confronted with a number of adverse events due to plant‐based products. Yet existing regulatory frameworks meant that they could not intervene as effectively as they would have wanted against such products. Policies on herbal medicines up to then had been generally in favour of the status quo, characterized by a relative tolerance to herbal medicines and in favour of self‐regulation, considered as a mark of UK liberal governance.^20^ The medicines regulator argued for an overhaul of the regulatory system that would address more effectively the kind of risks that it saw herbal products as posing, and approached it as a technical matter prompted by a concern to increase safety in the broad market for health products. The issue became more politicized once it reached Parliament, though less explicitly so in the House of Commons than in the House of Lords. In the former, debates on the regulation of herbal medicines and other non‐conventional therapies tended to focus at the time on how to prevent illegal claims made by herbal medicine manufacturers, rather than on the more substantive issue of which products should be allowed onto the market and under what conditions.^21^ There was an implicit acceptance of herbal medicine, at least as long as it remained confined to its sphere of practice, without challenging the medical profession. This stance had been cemented ever since the 1940s, when Aneurin Bevan made it clear to herbalists that inclusion in the National Healthcare Service (NHS) would be conditional on becoming subordinate to the medical profession.^22^ The tone in the House of Lords was very different. Safety issues were rarely the focus of the interventions; instead, the symbolic and socio‐cultural significance of herbal medicines was of greater concern. Indeed, many Lords were very supportive of complementary medicines more generally, and some even had active roles as patrons of ‘natural’ healing associations.^23^

From the early days of these debates, there were at least two narratives that fitted into two distinct visions of herbal medicines. On the one hand, health ministers and regulators such as the MCA were concerned about the safety of herbal medicinal products circulating in global markets, some of which were adulterated or contaminated with toxic plants or substances. On the other hand, many parliamentarians defended a particular idea about the place of herbalism in the UK. For some, it represented healing practices rooted in British folk culture and its multicultural society; for others, small herbal medicine producers and sellers represented the spirit of entrepreneurship of local communities. However, these views did not account for the fact that there were multiple actors who derived their practices from different sources of knowledge and operated differently. Herbalists and traditional healers, who were largely unregulated or self‐regulated via professional associations, supervised the herbal prescriptions after consultation and diagnosis, while the products sold by manufacturers could be bought by consumers without their supervision.

Outside Parliament, herbal and traditional healing associations also started to lobby for the protection of their profession and the materials upon which they relied. Notably, they sought to secure statutory recognition as legitimate healthcare professionals. Most agreed with the government that safety was an issue that needed to be addressed, but argued that this made their role as experts even more crucial.^24^ This role of herbalists as experts and the relationships that sustain the use of herbal medicines would ultimately be overlooked in regulatory debates, a point to which we return later in the article.

### 5.2 EU debates and the emergence of the Directive

At a broader regional level, debates about the safety of herbal medicines intensified throughout the 1990s. As in the UK, medicinal plants had increasingly become a matter of concern for EU regulators. Despite the density of medicine governance, medicinal plants had largely managed to escape its influence, often by being marketed as something other than medicines, even when consumers were using them as such. Throughout the 1980s and 1990s, the European Court of Justice struggled to police the existing legal boundaries between medicines, cosmetics, and food, which meant that numerous medicinal plants were travelling through EU markets without being subject to the checks that would more commonly be applied to health products.^25^ Individually, national regulators across the EU, such as the MCA in the UK, struggled to contain the proliferation of manufactured herbal products sold without clear guidance.^26^ Questions about the place of medicinal plants that did not fit the logic of the existing medicine regimes were often also part of wider debates about ‘complementary’ and ‘alternative’ medicine, and how to integrate it within the wider EU project, raising broader questions about the kind of understanding of health care that EU states shared.^27^ These were never resolved; instead, conversations focused on developing a specific regime for herbal products, made more urgent when many EU countries reported consumers experiencing severe health problems after using some herbal products, notably Chinese herbal diet pills. Different institutional and civil society actors joined the chorus calling for safeguards against the potential risks from plant‐based products. By 1995, the European Commission (EC) had begun investigating safety concerns around medicinal plant‐based products, including raw materials produced within the EU or imported from countries outside it.^28^ Civil actors’ reports and engagement also framed the matter of plants and their regulation as one of safety, which could be addressed using the same tools as those applied to medicines more generally.

As demands for stricter regulation started to be voiced at the EU level in the 1990s, marking the start of a process of harmonization, UK authorities were wary of the effects that this could have on the herbalist exemptions.^29^ Yet they could not avert an energetic public campaign against further harmonization of licensing laws instigated by the health food industry, herbal medicine manufacturers, and herbalists’ associations, and fronted by celebrities and politicians, who feared that any new regime would force legislators to remove existing exemptions.^30^ This was echoed by strong cross‐party responses against any measures that would limit access to existing herbal medicinal products. For the medicines regulator, these reactions suggested that its attempts to tighten the regulation of herbal medicines were unlikely to gain traction domestically. As well as turning its effort towards regulatory reform to the EU, it abandoned plans for direct regulatory reform at the domestic level, focusing instead on improving pharmacovigilance, bringing herbals into its surveillance system in 1996.^31^ By gathering data about the problem, the medicines regulator was able to alert the government to the need for further regulation, securing its support in EU forums, while establishing the legal monitoring of herbals as a technical matter of health and safety.^32^ Turning to the EU, and therefore shifting policy venues, had significant advantages, partly because both UK‐ and EU‐level regulatory authorities had been simultaneously preparing the ground to regulate herbal medicines and safeguard consumers’ health. Drawing on highly technical discourse and established expertise, the authorities were able to steer the course of the debates, convincing the Department of Health and Social Care that there was a problem that required action, and shield themselves from overtly politicized debates.^33^

The UK government embraced the framing of unregulated plants as risks to public health, which echoed the technical approach that the regulator had sought to initiate; the regulation of medicinal plants could be seen as another scientific and technological issue for which answers could be found using the kind of regulatory tools with which the field was already familiar. Indeed, the UK's highly influential role in EU institutions, particularly those entrusted with medicines laws and regulations, meant that it could steer the conversation in this direction, placing the regulation of medicinal plants firmly in the territory of safety, risk, and technological governance. However, in the back and forth between EU‐ and UK‐level debates, alternative narratives would continue to co‐exist concerning the significance of regulating herbal medicines, and, in the UK, how this would sit with the space offered by national legislation to herbalists. Within those narratives, the multiple meanings of ‘tradition’, and how they reflect different world‐making projects, became more visible.

### 5.3 The legal (re‐)emergence of traditional herbal medicines

The first reference to ‘traditional use’ appeared in a debate in the House of Commons in 1994, in the context of a proposed reform to harmonize medicines laws in the EU.^34^ According to Thomas Sackville, the Secretary of State for Health, medicines made under the current exemptions used ‘a number of traditional processes’ that made it very unlikely that the European Court of Justice would consider them to be within the scope of the pharmaceutical medicines regime. He concluded that ‘the position of herbal medicines in the UK was … safeguarded under EC law’.^35^ However, once the UK government began to lobby for a Traditional Herbal Medicinal Products Directive in the late 1990s, ministers no longer assured Parliament that the status quo would remain. Instead, Tessa Jowell, Sackville's successor, told members of Parliament (MPs) in 1999 that the exemptions for unlicensed medicines did not offer ‘protection to the public against low‐quality and unsafe unlicensed herbal remedies which are known to reach the UK market’.^36^ Marking a shift in policy, she also stressed the government's intention to find workable solutions to protect consumers from dangerous and ineffective herbal medicines and support ‘responsible business and practitioners to operate effectively’.^37^ The boundaries between what the herbalist exemptions were supposed to protect and what the new Directive was seeking to do would remain at the core of ongoing debates for years to come. Tensions around the interface between the new regulations and herbalist practices escalated throughout the debates on the Directive, and after its implementation. Through these debates and the implementation process, it became clearer that herbal medicines on the one hand and their regulations on the other represented contrasting ideas of the kind of world that needed protecting and the role that herbals should play in the UK's futures. ‘Tradition’ with respect to medicines became a slippery term mobilized in different ways, and for different purposes, to put forward socio‐political priorities. For opponents of the new regulatory regimes in the making, herbal medicines and their regulation could not be reduced to a technical matter, subsumed in a purely socio‐technical future. Their arguments featured both in debates on the new regulations per se and in cross‐references to herbal medicines and their protection in tangential policy discussions.

For example, during the 1994 campaign in support of protecting the existing market for herbal medicines, some argued that reforming medicines law to encompass herbal medicines could threaten multicultural health care. Changing the availability of herbal medicines would imperil the practices upon which some communities had relied for generations, and would therefore be a matter of cultural policy as well as health legislation. A cross‐party motion insisted on the value of these practices to ethnic communities from former British colonies. Specifically, the motion noted ‘the valuable contribution made to the health of millions of British subjects, over many centuries, by the use of herbal remedies’ and argued that the potential loss of the herbal medicines marketed in the UK would damage ‘those who practise the systems of medicine favoured by many in the Asian, Caribbean and Chinese communities’.^38^ By contrast, during the debates on the Directive in the UK Parliament, this concern for communities was overshadowed by a more explicit ‘nostalgia for Empire’, particularly when the same actors were elsewhere voicing opposition to the EU project as a whole on similar grounds.^39^ Alongside protecting multicultural healing (regardless of the motivations), others emphasized the need to preserve the more localized heritage that folk herbalism represented, placing a different kind of identity and history at the core of their concerns. In those discourses, herbalism (beyond herbal medicines per se) needed to be protected as a set of traditional practices whose disappearance would have socio‐cultural as well as medicinal impacts.

As these debates intensified, the concept of ‘traditional herbal medicines’ occupied centre stage in the EU by becoming a term on which the Directive would hinge. In official documents at the EU level, the first mention of ‘traditional use’ appeared in 1999, in a report of the Pharmaceutical Committee (now the Human Pharmaceutical Committee).^40^ It is worth saying that though herbal medicines had already been on the radar of the EC and the European Medicines Agency for several years, the terminology used had been ‘herbal medicine’, not ‘traditional herbal medicine’.^41^ The EC proposed a draft, which was followed up by further debates in the Pharmaceutical Committee, and at a working‐group level.^42^ The idea of having a special category for ‘traditional herbal medicines’ framed the terms of the debates; once the idea that herbal medicines should be regulated more strictly had made its way into EU arenas, it was also agreed that they should not all be subject to the stringent checks and controls imposed on pharmaceutical products (including the need to present the results of clinical trials, which were seen as unnecessary and creating too heavy a burden for the kind of industries that produced some herbal products). In particular, the proposal was that those herbal products that had a long history of traditional use should be offered a particular, lighter‐touch regime. When the European Commissioner presented the Directive to the European Parliament in 2002, it became clear that the technical framing agreed in the Pharmaceutical Committee had taken hold. With herbal medicines framed as risky products in need of technical surveillance, ‘tradition’ represented a pragmatic way to ensure protection for consumers. In the words of the Commissioner, the purpose of the Directive was to ‘guarantee a high level of health protection’.^43^

Some delegations pointed out that this framing simplified or overlooked important aspects of the debate on regulating medicinal plants and did not sufficiently acknowledge the significance or complexity of traditional medicines. For example, the Swedish and Irish delegations argued that more respect should be paid to the plurality of traditions in Europe, understood as knowledges and praxis around medicinal plants – a view that aligned more with that of the World Health Organization (WHO).^44^ If these concerns echoed some of those that had been brought up during the UK debates (and would continue to be so as the Directive was adopted and later implemented), they were ultimately abandoned, as the pragmatic approach to health and safety was prioritized.

### 5.4 Redefining traditional herbal medicines

The Directive adopted a technical approach to ‘tradition’ characterized by a certain pragmatism.^45^ Its provisions, including the definition of ‘traditional use’, turned a socio‐political process into a regulatory technical solution.^46^ It did not define ‘tradition’ as a set of particular skills or a body of knowledges; instead, ‘tradition’ was defined on the basis of time‐based criteria that equated ‘traditional use’ with ‘well‐established’ use documented over time.^47^ Those seeking approval for products under the simplified registration system that was being established would have to demonstrate that the products had been used in the EU for 15 continuous years or 30 years anywhere elsewhere in the world.^48^ Significantly, ‘tradition’ became the key to a differential regime for herbal medicines that was nonetheless located within the broader pharmaceutical regulatory landscape. Yet, by embracing a technical and bounded definition of ‘tradition’, the Directive also produced a new definition of ‘herbal medicine’ that was disconnected from broader cultural debates about different approaches to healing and medicine.

The Directive also set out criteria that products needed to meet to be approved under the new regime. The use and redefinition of the word ‘traditional’ itself sidelined other debates and interpretations. Furthermore, the criteria were based on a specific idea of what traditional herbal medicines were and how they should be used. At the outset, traditional herbal medicines were imagined to be sufficiently safe to be sold without the need for professional oversight; they would be, like over‐the‐counter products, destined for self‐administration. As pointed out by the Economic and Social Committee of the EC, self‐medication, together with growing demand and supply of herbal medicines through ‘alternative outlets’ (meaning herbalists, health food shops, and mail orders on the internet), was the concern that led to the development of the Directive.^49^ This focus on self‐medication meant that the Directive restricted the kinds of therapeutic claims that could be attached to traditional herbal medicines so that they should be limited to minor conditions, or some chronic conditions, that did not require professional oversight. However, the suggestion that herbal medicines could be dissociated from expert knowledge contradicted how others, including some of the groups that had been represented in the UK Parliament, for example, envisaged herbal medicines; in other understandings of traditional herbalism, the value of herbal remedies is rooted in the relationships that surround their use. In this view, rather than being inherently powerful as health devices, plants are effective only in conjunction with the accompanying knowledge, rituals, and practices.^50^ This does not mean that they are mere placebos, but that the human component and expertise on how to mix plants are variables affecting their efficacy.

This initial assumption that traditional herbal medicines as defined by the Directive should be suitable for self‐medication had implications for both the composition of products and their presentation. Regarding the composition and manner of use, the Directive specified that products are to be used in accordance with a specified strength and dosage, and mode of administration (oral, external, or inhaled). The requirement of specific dosage and strength meant that they would need to be produced and processed in ways that would make them look more clearly like pharmaceuticals, rather than closer to raw plants, for which dosage is more approximate. The Directive also required that the presentation of these products should conform to their intended purpose as over‐the‐counter medicines for minor conditions, distinguishing them from prescription‐only pharmaceuticals, which require medical supervision. Overall, ‘tradition’ became a signifier that a product was safe enough to be used as an over‐the‐counter medicine. However, in that process, herbal medicines were made to adapt and take the appearance of over‐the‐counter medicines; they needed to shed the plurality of identities that they had had until then. For example, to be registered, a traditional herbal medicine must be appropriately labelled; both external labels and inner leaflets should state the identity of the product. As with medicines, the inner leaflet must state clearly the mode of administration and a specified strength and dosage. Furthermore, much like medicines, the external labelling and advertising must reflect the category to which it belongs; any claims to efficacy on the packaging must make clear that they are based on long‐standing use rather than clinical evidence. Unlike herbal preparations made by herbalists, these products are standardized – made to resemble each other, in substance and presentation – for the sake of quality control.^51^ Meanwhile, the Directive strongly linked quality to existing industry standards for assessing the safety of products, including physicochemical, biological, and microbiological tests. To facilitate registration applications and the harmonization of traditional herbal medicines, the Directive also established the Herbal Medicinal Products Committee (HMPC), responsible for creating monographs and lists of herbal substances across the EU. These monographs, which can be referenced in applications, contain scientific evaluations of herbal medicinal products based on scientific data (available clinical trials) or historic use (bibliographic references).^52^

All in all, even though herbal medicines were offered a particular regime on the grounds of their ‘traditional’ nature, one effect of this new regime was to fold them into a more explicitly socio‐technical frame. By contrast, less attention was paid to other components of the long‐standing practices that have sustained the use of particular plants, such as herbalist knowledge, relationships with users, and the broader networks that have characterized some healing practices. Ironically, these may be seen by herbalists as more crucial to ensuring safety than industrial transformation or standardization. These concerns were expressed in the UK by those who held a different understanding of traditional herbalism and its stakes. From their perspective, the Directive brought about a ‘pharmaceuticalized’ version that folded traditional practices into a set of safety requirements and norms deriving from a medicine regime.^53^ They opposed the reduction of tradition to something quantifiable, and the standardization of its materials beyond recognition, because this denied herbalism its distinction from biomedicine, effacing the socio‐political aspirations that animate their everyday practices. By contrast, the Directive fitted a vision of the future resting on techno‐scientific means, in which products should be classified through the particular tests and mechanisms of technoscience and regulated through the type of bureaucratic procedures that support them. In this version of the world, a certain level of uniformity and standardization is required, and this can be achieved by detaching traditions from their cultural basis, to focus instead on material embodiments. A traditional herbal medicine is transformed into a consumer object: a clean box with only the brand name, directions for use, ingredients, and warnings. However, by restricting registered herbal medicines to self‐consumption, the Directive silently disregarded the authority of healers and their role in making traditions; instead, the specific provisions reasserted the products’ connection to the professional medical world, inscribing a pharmaceutical imaginary onto the materiality of the package. Despite some compromises, the Directive did not deviate from existing frameworks that have characterized the EU regulation of pharmaceutical drugs and science, embodied in long‐standing practices of cataloguing, translating, and transforming plants’ biochemical potentials through socio‐technical intermediaries such as laboratories, clinical tests, sanitized machines, standardized manufacturing processes, and pharmaceutical monographs. On the contrary, it embedded existing socio‐technical imaginaries about the role of law, medicine, and technology in its specific provisions.

## 6 EVERYDAY FRICTIONS AND ALTERNATIVE VISIONS OF TRADITIONAL HERBAL MEDICINES

Some of the limits of this imaginary of traditional herbal medicines, as determined by the Directive, soon appeared in responses at the UK level. Those who had previously shown their attachment to the herbalist exemptions and had argued that medicinal plants were cultural artefacts in need of particular consideration were concerned by the implications of the Directive. With support for folk practices sometimes overlapping with a sense of nationalism, and despite the fact that the EU's approach was influenced by the UK, Eurosceptic parliamentarians were quick to reframe the issue as one of undue EU intrusion into national matters. This resulted in an attempt to compromise in the process of implementation at the UK level, and to protect the space covered by the herbalist exemptions – or at least what the UK legislator imagined as being this space.

The possibility of compromising to protect folk herbalism from the EU's regulation of herbal medicines had been floated before. In the context of the 1994 campaigns in defence of herbal products, as we saw, some had suggested that the EU's focus on industrially produced herbals meant that the Directive was unlikely to affect herbalist practice.^54^ Recalling exchanges with government officials, Michael McIntyre, former head of the European Herbal and Traditional Medicine Practitioners Association, said that UK authorities had suggested that some products made under particular licensing exemptions would fall outside the scope of the EU legislation because they were ‘traditional, not industrially produced’.^55^ However, as the explicit framing of the Directive centred on ‘traditional’ products, it became clear that such a guarantee could not be given with any certainty. Later that year, the MCA launched a consultation document on the harmonization of UK medicines that confirmed that only ‘industrially produced’ medicines needed to be licensed, limiting any impact on existing exemptions for herbalists’ products.^56^ This thin line would continue to be negotiated as the Directive was implemented. It rested on a clear distinction between two versions of herbal medicine: one close to that embedded in the Directive, where herbal medicines would essentially be manufactured, standardized products, and the other, where herbal medicines would be used as raw products, in the context of an arguably romanticized version of folk herbalism. When the Human Medicines Regulations of 2012 incorporated the registration scheme, which put into effect the provisions of the Directive, it reflected this distinction between herbal medicines that were industrially made and those that were not. Specifically, it set out that anyone could still make herbal medicines for someone else as long as they were ‘not “manufactured” or assembled on a large scale or by an industrial process’.^57^

Once the new rules came into force, however, it became clear that the visions of herbalism embedded in the law did not reflect some practices or all ‘traditions’. In effect, the legislation adopted the kind of static vision of ‘tradition’ that critics have long sought to unpack and challenge. Though herbalists were invited to participate in a series of consultations and contested the lack of consideration for what they perceived as ‘traditional’ in their everyday practice, what stood unchallenged in the law‐making process was the image of a folk herbalist, dealing essentially with raw plants on a very small scale. The law overlooked the fact that herbalism, like all healing practices, is a living tradition; practices have multiplied and hybridized over the years, and those who had been excluded from the vision proposed by the EU would not all fit into the alternative model of ‘tradition’ adopted by the UK legislator. Herbalists, who had sought to be heard since the issue of reinforcing the regulation of medicinal plants had come up in the UK, but with little success, were frustrated by the outcome, and at finding their practices once again erased or misread.^58^ In particular, the new legislation had a striking blind spot: it failed to recognize that even small‐scale herbalist practice had long been dependent on some herbal products that could arguably be seen as ‘manufactured or assembled’, without reaching the level of industrialization and standardization implied by the Directive, nor adopting its preference for self‐medication. This kind of practice had been fostered and enabled by the previous exemptions offered under the 1968 Medicines Act. It rested on relationships that had become an intrinsic part of the everyday practice of herbalism, and of a different version of what contemporary ‘traditional herbalism’ could look like.^59^

Alongside this issue, other inadequacies of the Directive's redefinition of traditional herbal medicine emerged in the course of its implementation. One of the most contentious issues was that the Directive and its implementation disadvantaged non‐European traditions, particularly through the provision requiring products to demonstrate medicinal use for at least 15 continuous years in the EU or 30 elsewhere, and those that barred the use of substances derived from animals or minerals.^60^ In the UK, this came to a head in 2002, when the exclusion of traditional Asian medicines drew strong condemnation in a motion signed by 219 MPs, who urged the government ‘to renegotiate the main provisions of this flawed Directive’ because it risked excluding existing popular herbal remedies.^61^

Ayurvedic and traditional Chinese medicine practitioners were particularly affected by these issues and the exaggerated distinction between manufactured products and other kinds of herbal medicines. Their practices relied heavily on complex manufactured formulations (common in both Ayurveda and traditional Chinese medicine) or on dispensaries (more common in traditional Chinese medicine) and needed significant adjustments to meet the new regulatory requirements. Traditional Chinese medicine and Ayurvedic associations justified the use of third‐party suppliers because they could be trusted to offer high‐quality manufactured products. Some of the preparations that they used had already been ‘modernized’ through China and India's regulatory trajectories, which for practitioners made them ‘safer’ than what they could individually produce in the UK.^62^ However, even these modernized versions could not be approved through the traditional herbal medicine regime in the EU because some of the formulas contained animal or mineral substances. When the new regulations came into effect, the medicines used by Ayurvedic and traditional Chinese medicine practitioners that had flourished under previous herbalist exemptions were pushed to the fringes of legality. Even where their proprietary formulas were eligible for registration under the traditional herbal medicine registration scheme, practitioners were limited in how they could use such formulas in their practice, since the Directive framed traditional herbal medicines as those intended for self‐medication purposes, rather than to be used as part of a consultation or prescription. Caught in this double bind, Ayurvedic and traditional Chinese medicine stakeholders felt that the legal space in which to practice had shrunk. Even if this was not the intention, one effect of the Directive was to make practitioners’ role uncertain, excluded from its model of traditional herbal medicines, in which healers would not be needed anymore.

In response to this new sense of precarity, practitioners have adopted novel ways of providing mixed herbs. For example, some have begun selling single ingredients and telling patients to mix them themselves to produce a particular Ayurvedic medicine, thereby avoiding the formal manufacturing process. Others have retreated into the grey areas between foods, food supplements, and plant‐based medicines, finding safety in relabelling their practice away from the new regimes that apply to herbal medicines. As a traditional medicine association interviewee noted,

People come and see them and patients are fully aware that this medicine is not available, … and … you were always told … it's not treated as a medicine. They are all food supplements. Nothing is given as a pill [with the words] ‘Here is a medicine’. None of us could say ‘We are going to cure something’. The result is, it does cure something. We can't say that. We can't put that label, because we are not allowed to. That is what it is like. It's made it sort of slightly underground, but not so much, but it's finding clever ways around it.^63^

Western herbalists were also affected by the ban on requesting third parties to manufacture bespoke herbal medicines, and have had to adjust their practice in similar ways, capitalizing on the possibilities offered by individual consultations and negotiating the boundaries between food products and the exemptions still allowed for herbal medicines prepared after a consultation.^64^ As the new regulations made some of the processes that they had used before illegal, some herbalists have adopted the vision of folk herbalism embedded in the new exemptions, celebrating the local sourcing of plants, foraging, and the use of raw materials added into food or self‐made herbal mixtures.^65^ In response to the Directive's redefinition of traditional medicines as industrial versions of themselves, they hardened their opposition towards the commercialization and exploitation of herbal medicines, on environmental and ethical grounds. The underlying contrast between the imaginaries deployed by regulators and herbalists has become starker as a consequence. While regulators made the future of traditional herbal medicines dependent on their adherence to standardized modes of production, herbalists grounded their engagement with medicinal plants in their resistance to an all‐industrial future, replaying the history of early modern Western herbalists, who seized upon herbs as an alternative to potent industrialized medicines.^66^ Moreover, these herbalists reaffirmed herbalism as being essentially a popular form of medicine that should be available to all regardless of skills or resources, and lamented the risk of a growing dependence on ready‐made products.

This has directly affected herbalists’ response to the law, and indeed their broader positioning vis‐à‐vis the law. Some herbalists have not only resisted the models that underpin the new regulations but their substance too. For most, this has not taken the form of explicit resistance, but instead of careful everyday negotiation and avoidance, challenging the underlying world‐making intention of the law by tinkering with its application. The law has not lost all of its authority or significance, but it has lost some of its credibility, making some breaches acceptable. For example, some of our interviewees acknowledged that some herbal medicines that they made for specific minor conditions (such as skin creams or cough syrups) transgressed the new regulations insofar as they were not made for a specific patient. One continued to sell products at open‐air markets that are technically more akin to over‐the‐counter herbal medicines than to tailored products made in the context of a consultation and for that reason requiring authorization from the medicines regulator. Illegal acts such as this prompted warnings from the MHRA. Yet, for many community herbalists, selling their over‐the‐counter herbal medicines had been a way to get known and build trust with people who could become potential repeat customers.^67^ This tactic helped to build relations where the herbalist ‘connects’ patients to the healing plants, framing their use within a specific epistemology of healing.^68^ Others did not sell these remedies, but instead donated them to people through herbal free clinics and refugee camps in France, arguably also supplying these products illegally but fulfilling an ethos of sharing medicines where the state has failed to do so.^69^ Some also negotiated boundaries, notably those of what may constitute a ‘one‐to‐one consultation’. One herbalist explained that they tried to comply with the requirement for one‐to‐one consultations by giving generic preparations rather than bespoke products after asking customers a few questions.

Overall, the Directive and its implementation have unsettled pre‐existing herbalist practices that did not fit into the socio‐technical imaginaries embedded in the new regulatory framework. By reimagining traditional herbal remedies as a particular category of manufactured products, disconnected from healing relations, the new registration system pushed those who performed an alternative form of herbalism to the fringes of the law. While the Directive turned ‘tradition’ into a placeholder for safety, and industrial medicines co‐opted the label, herbalists in the UK have also adopted less industrial and more artisanal and rudimentary ways to go undetected or rearranged how they source materials. Some herbalists have chosen to go ‘back to basics’, simplifying their medicines by using fewer herbs or adapting their methods to comply with the new imaginaries of tradition embodied in the regulations.^70^ Seeing some of their long‐standing practices erased by the regulatory process, they have had to reinvent their sense of ‘tradition’. Sometimes, this has meant hardening their stance as a form of resistance to an all‐industrial future; at other times, it has entailed redefining their role. The Directive has also drawn new distinctions between different types of material product, encouraging manufacturers to adapt their products to pharmaceutical industry standards, while herbalists have embraced practices embedded in folk medicine imaginaries of ‘tradition’, adjusting their remedies accordingly. As some herbalists have retreated to folk and local imaginaries, identifying their ‘tradition’ with the ‘indigenous’ herbalism of the UK, and other traditions, such as Ayurveda, have dispensed with the use of ‘modern’ technologies in the manufacture of their medicines, the effect in both practices has been a further blurring of the categories to which herbal medicines belong.^71^ While the new UK regulations gave the appearance of having neatly classified herbal medicines as either ‘home‐made’ or industrial, and as either ‘modern’ or ‘traditional’, the boundaries continue to be traversed.^72^ Ultimately, and echoing Craig, these distinctions – between ‘traditional’ and ‘modern’, between ‘global’ and ‘local’ – are performative and often overlap, creating ‘multiple medical realities’.^73^ Ironically, the displacements also reveal how this multiplicity has resulted from the ongoing attempts to make herbals a more acceptable form of medicine, but only if aligned with biomedical imaginaries and their embedding in law.

## 7 CONCLUSION

The story of the emergence of the EU's Traditional Herbal Medicinal Products Directive and how it has translated into everyday practice in the UK is also a story about the relationship between imaginaries of medicine, and socio‐legal relationships. While competing imaginaries of what herbal medicines are about, and what kinds of social world they are best suited to, have co‐existed for decades, the Directive has embraced one particular vision of the conditions under which traditional herbal medicines should fit into contemporary societies, thereby redefining them both rigidly and narrowly. It has produced a partial view of ‘tradition’ in herbal medicine that excludes the different experiences of ‘tradition’ and modes of making herbal medicines that others have sustained through their practices. In doing so, it has appropriated and rewritten the meaning of ‘tradition’, cutting its ties to numerous social and historical relationships and adapting it to the demands of industrial and techno‐scientific modes of production aligned with contemporary healthcare practice. Efforts in the UK, during the process of implementation, to recognize the existence of a different imaginary of ‘tradition’ in herbal medicines were only partly successful. While national legislation carved out a space in which herbalists could continue some of their pre‐existing practices, it failed to recognize traditions as living practices. Instead, the industrial model of ‘tradition’ embedded in the Directive has helped the reorganization of the field of herbal medicines by embracing a reductive vision of herbalism based on a re‐imagined past, one that is slightly out of touch with contemporary conceptions of herbalist traditions in the UK.

Many herbalists have found themselves excluded from both of those legal translations of traditional herbalism, and many of their practices have either become illegal or fallen into a legal grey area. This has affected both their practice and their relationship to the law. While some were initially open to legal reform, to protect themselves and their customers, their exclusion from the enactment of the law has made them more sceptical. Their response has been one of everyday negotiation, to try to make their practice arguably fit within the law, while also being aware that some of their activities may be bending the rules. Without openly breaking the law, they may stretch its boundaries in everyday practice. At the same time, this negotiation with the law is a matter of survival, for them as professionals, but maybe more importantly for the visions of herbalism that they seek to sustain. Noting the blindness of law to their practices, some have chosen to make their vision of herbalism more radical, opposing more openly through their daily practices the kind of industrial future for herbalism that the Directive seems to have sketched. In this way, practices that challenge the boundaries of the law are also acts of resistance, sustaining world‐making practices in which herbal medicines can take different forms, distinct from their industrial incarnations. As the letter of the law is worked around or negotiated with, its spirit has affected its legitimacy and the legal consciousness of those actors whom it has newly excluded from its redefinition of traditions, despite their role in sustaining herbalism over the years. At the same time, there are limits to their ability to resist, and the effects of the law are undeniable. Some forms of medicine making that had developed in the UK are no longer sustainable, and practices have had to adapt to new difficulties. Even where the law is seen as lacking legitimacy, its world‐making powers are recognized. In crossing legal boundaries, herbalists also perform acts of resistance to a particular kind of socio‐technical future.

## Footnotes

1 B. Griggs, *New Green Pharmacy: The Story of Western Herbal Medicine* (1997).

2 P. Chakrabarti, *Materials and Medicine: Trade, Conquest and Therapeutics in the Eighteenth Century* (2010).

3 S. Bhamra et al., ‘The Use of Traditional Herbal Medicines amongst South Asian Diasporic Communities in the UK’ (2017) 31 *Phytotherapy Research* 1786; G. Green et al., ‘“We Are Not Completely Westernised”: Dual Medical Systems and Pathways to Health Care among Chinese Migrant Women in England’ (2006) 62 *Social Science & Medicine* 1498.

4 L. Teng et al., ‘Characteristics and Practices of Traditional Chinese Medicine Retail Shops in London: A Cross‐Sectional Study Using an Observational Approach’ (2015) 173 *J. of Ethnopharmacology* 318; M. Banerjee, *Power, Knowledge and Medicine: Ayurvedic Pharmaceuticals at Home and in the World* (2009).

5 Herbal Medicines and Practitioners Regulation Meeting with David Walker DCMO, *Small Group Meeting Minutes* (2014), at <https://assets.publishing.service.gov.uk/government/uploads/system/uploads/attachment_data/file/417778/Small_Group_Meeting_minutes.pdf>.

6 E. MacLennan and B. A. Pendry, ‘The Evolution of Herbal Medicine as an Unorthodox Branch of British Medicine: The Role of English Legislation from 1914 to the Present Day’ (2011) 1 *J. of Herbal Medicine* 35.

7 J. Stone, *Complementary Medicine and the Law* (1996).

8 J. P. Gaudilliere and L. Pordie, ‘The Reformulation Regime in Drug Discovery: Revisiting Polyherbals and Property Rights in the Ayurvedic Industry’ (2014) 8 *East Asian Science, Technology and Society: An International J*. 573; A. Winterbottom, ‘Becoming “Traditional”: A Transnational History of Neem and Biopiracy Discourse’ 36 *Osiris* 262.

9 H. Tilley, ‘Traditional Medicine Goes Global: Pan‐African Precedents, Cultural Decolonization and Cold War Rights/Properties’ (2021) 36 *Osiris* 132.

10 For a more focused engagement with rhetoric and medical law, see J. Harrington, *Towards a Rhetoric of Medical Law* (2018).

11 M. Flear and R. Ashcroft, ‘Law, Biomedical Technoscience, and Imaginaries’ (2021) 8 *J. of Law and the Biosciences* 1, at <https://doi.org/10.1093/jlb/lsaa088>.

12 S. Jasanoff and S.‐H. Kim, ‘Containing the Atom: Sociotechnical Imaginaries and Nuclear Power in the United States and South Korea’ (2009) 47 *Minerva* 119, at 120.

13 M. Flear, ‘Regulating New Technologies: EU Internal Market Law, Risk and Sociotechnical Order’ in *New Technologies and EU Law*, ed. M. Cremona (2017) 74.

14 A. Mahalatchimy et al., ‘Framing and Legitimating EU Legal Regulation of Human Gene‐Editing Technologies: Key Facets and Functions of an Imagery’ (2021) 8 *J. of Law and the Biosciences* 1, at <https://www.ncbi.nlm.nih.gov/pmc/articles/PMC8366714/>.

15 E. Grabham, *Brewing Legal Times: Things, Form and the Enactment of Law* (2016).

16 Mahalatchimy et al., op. cit., n. 14.

17 S. Halliday, ‘After Hegemony: The Varieties of Legal Consciousness Research’ (2019) 28 *Social & Legal Studies* 859; D. Cowan, ‘Legal Consciousness: Some Observations’ (2004) 67 *Modern Law Rev*. 928.

18 See for comparison E. D. Fritsvold, ‘Under the Law: Legal Consciousness and Radical Environmental Activism’ (2009) 34 *Law and Social Inquiry* 799; S. Halliday and B. Morgan, ‘I Fought the Law and the Law Won? Legal Consciousness and the Critical Imagination’ (2013) 66 *Current Legal Problems* 1.

19 A. Littoz‐Monnet, ‘The Role of Independent Regulators in Policy Making: Venue‐Shopping and Framing Strategies in the EU Regulation of Old Wives Cures’ (2014) 53 *European J. of Political Research* 1.

20 Hansard, HC, vol. 82, cols 528–530W (11 July 1985), at <https://api.parliament.uk/historic‐hansard/written‐answers/1985/jul/11/natural‐medicines#S6CV0082P0_19850711_CWA_291>.

21 Hansard, HC, vol. 251, col. 357W (8 December 1994), at <https://api.parliament.uk/historic‐hansard/written‐answers/1994/dec/08/herbal‐medicines#S6CV0251P0_19941208_CWA_401>; Hansard, HC, vol. 251, col. 135 (6 December 1994), at <https://api.parliament.uk/historic‐hansard/commons/1994/dec/06/herbal‐medicine#S6CV0251P0_19941206_HOC_80>; Hansard, HC, vol. 152, col. 193W (6 December 1994), at <https://api.parliament.uk/historic‐hansard/written‐answers/1994/dec/06/herbal‐remedies#S6CV0251P0_19941206_CWA_397>.

22 MacLennan and Pendry, op. cit., n. 6.

23 Hansard, HL, vol. 518, cols 1400–1436 (9 May 1990), at <https://api.parliament.uk/historic‐hansard/lords/1990/may/09/medicine‐complementary‐and‐conventional>; Hansard, HL, vol. 568, cols 132–186 (10 January 1996), at <https://hansard.parliament.uk/Lords/1996‐01‐10/debates/e87c21ad‐abe0‐4ff2‐bf64‐bf3e2782bff2/Non‐ConventionalMedicalTreatment>.

24 M. McIntyre, ‘Statutory Regulation: A Legislative Basis for Herbal Practice’ (2011) 1 *J. of Herbal Medicine* 30.

25 B. Van der Meulen, ‘Medicinal Claims: Prohibition, Enforcement and Delineation: Food in Fact but Medicine in Law’ (2017) 5 *European Food and Feed Law Rev*. 392; P. Diaz Peralta et al., ‘Regulating the Risks from Traditional Herbal Medicine Resources and Herbal Supplements in the EU: The Borderline Products’ (2016) 4 *European Food and Feed Law Rev*. 274.

26 J. Barnes et al., *Herbal Medicines* (2007).

27 A. Rogers, ‘Europe Fails to Unite Alternative Medicine’ (1997) 349 *The Lancet* 1679; P. Lannoye, *Report on the Status of Non‐Conventional Medicine* (1998) Committee on the Environment, Public Health and Consumer Protection, PE 216.066/fin, at <https://www.europarl.europa.eu/doceo/document/A‐4‐1997‐0075_EN.html>.

28 Council Resolution of 20 December 1995 on Medicinal Plant Preparations [1995] OJ C350/6, at <https://eur‐lex.europa.eu/legal‐content/EN/TXT/PDF/?uri=CELEX:31995Y1230(05)&from=FR>.

29 Banerjee, op. cit., n. 4.

30 MacLennan and Pendry, op. cit., n. 6.

31 MHRA, *The Safety of Herbal Products* (2002), at <https://hfnet.nibiohn.go.jp/usr/kiso/ninpu‐herb/HerbalsSafetyReportJuly2002_Final.pdf>.

32 Littoz‐Monnet, op. cit., n. 19.

33 Id.

34 Specifically, Regulation SI 3144 (1995) implemented in the UK the full range of controls in the first EU pharmaceutical medicines Directive. Council Directive 65/65/EEC of 26 January 1965 on the Approximation of Provisions Laid Down by Law, Regulation or Administrative Action Relating to Proprietary Medicinal Products [1965] OJ L22/369, at <https://eur‐lex.europa.eu/legal‐content/EN/TXT/?uri=CELEX%3A31965L0065>.

35 Hansard, HC, vol. 250, col. 86W (22 November 1994), at <https://api.parliament.uk/historic‐hansard/written‐answers/1994/nov/22/herbal‐remedies#S6CV0250P0_19941122_CWA_144>.

36 Hansard, HC, vol. 328, col. 427W (26 March 1999), at <https://api.parliament.uk/historic‐hansard/written‐answers/1999/mar/26/herbal‐medicines#S6CV0328P0_19990326_CWA_66>.

37 Id.

38 Hansard, HC, ‘Early Day Motion 14’ (16 November 1994), at <https://edm.parliament.uk/early‐day‐motion/11855>.

39 C. Koegler et al., ‘The Colonial Remains of Brexit: Empire Nostalgia and Narcissistic Nationalism’ (2020) 56 *J. of Postcolonial Writing* 585.

40 Pharmaceutical Committee, *Information on the Outcome of the 47th Meeting, 15–16 April 1999* (1999), at <https://ec.europa.eu/health/system/files/2016‐11/pc47inf_en_0.pdf>.

41 Pharmaceutical Committee, *Information on the Outcome of the 45th Meeting, 16–17 March 1998* (1998), at <https://ec.europa.eu/health/system/files/2016‐11/pc45inf_en_0.pdf>.

42 The Human Pharmaceutical Committee is a body where the EC, the European Medicine Agency, and members interact on issues of policy and implementation of the EU pharmacy laws. See also Pharmaceutical Committee, *Information on the Outcome of the 48th Meeting, 27–28 September 1999* (1999), at <https://ec.europa.eu/health/system/files/2016‐11/pc48inf_en_0.pdf>; Pharmaceutical Committee, *Information on the Outcome of the 50th Meeting, 21–22 September 2000* (2000), at <https://ec.europa.eu/health/system/files/2016‐11/pharcom50_en_0.pdf>.

43 European Commission, ‘Communication from the Commission to the European Parliament According to the Second Subparagraph of Article 251 (2) of the EC Treaty Concerning the Common Position of the Council on the Adoption of a Directive of the European Parliament and of the Council Amending, as regards Traditional Herbal Medicinal Products, Directive 2001/83/EC on the Community Code Relating to Medicinal Products for Human Use’ (2003) SEC/2003/1247 final, COD 2002/0008, para. 2, at <https://eur‐lex.europa.eu/legal‐content/EN/TXT/HTML/?uri=CELEX:52003SC1247>.

44 Pharmaceutical Committee, op. cit. (2000), n. 42.

45 MHRA, *Explanatory Memorandum to the Medicines (Traditional Herbal Medicinal Products for Human Use) Regulations 2005* (2005) para. 7.2, at <https://www.legislation.gov.uk/uksi/2005/2750/pdfs/uksiem_20052750_en.pdf>.

46 R. Jütte et al., ‘Herbal Medicinal Products: Evidence and Tradition from a Historical Perspective’ (2017) 207 *J. of Ethnopharmacology* 220.

47 The Economic and Social Committee noted that the Directive addressed ‘herbal medicinal products that have been well‐established over time (defined as “traditional”)’. See Economic and Social Committee, ‘Opinion of the Economic and Social Committee on the “Proposal for a Directive of the European Parliament and of the Council Amending Directive 2001/83/EC as Regards Traditional Herbal Medicinal Products”’ [2003] OJ C61/9, at <https://eur‐lex.europa.eu/legal‐content/EN/TXT/HTML/?uri=CELEX:52002AE1008&from=EN>.

48 Acceptable sources of bibliographic evidence of ‘traditional use’ included: toxicological and medical databases, reports, or monographs published by recognized sources such as the WHO and the European Scientific Cooperative on Phytotherapy; handbooks of medicine, phytomedicine, and herbal medicine; national formularies; monographs in the EU pharmacopoeia or official national pharmacopoeia; and post‐marketing studies. See C. Anquez‐Traxler, ‘The Legal and Regulatory Framework of Herbal Medicinal Products in the European Union: A Focus on the Traditional Herbal Medicines Category’ (2011) 45 *Therapeutic Innovation & Regulatory Science* 15.

49 Economic and Social Committee, op. cit, n. 47. See also MHRA, op. cit., n. 31.

50 S. A. Langwick, ‘A Politics of Habitability: Plants, Healing and Sovereignty in a Toxic World’ (2018) 33 *Cultural Anthropology* 415; D. Gibson, ‘KhoiSan Indigeneity and Entangled Becomings with Kanna, a Teacher Plant’ (2019) 5 *Catalyst: Feminism, Theory, Technoscience* 1.

51 Council Directive 2002/24/EC of the European Parliament and of the Council of 31 March 2004 Amending, as Regards Traditional Herbal Medicinal Products, Directive 2001/83/EC on the Community Code Relating to Medicinal Products for Human Use [2004] OJ L136/85, at <https://eur‐lex.europa.eu/legal‐content/en/ALL/?uri=CELEX%3A32004L0024>.

52 Barnes et al., op. cit., n. 26.

53 E. Vanmarie, *Re‐Presenting Herbal Medicine as Phytotherapy: A Strategy of Professionalisation through the Formation of a ‘Scientific’ Medicine* (2002).

54 McIntyre, op. cit., n. 24.

55 McIntyre argued that government plans to locate the European Medicines Agency in London would be frustrated by a parliamentary veto unless the government committed to protecting the availability of herbal medicines, but civil servants believed that the argument that unlicensed herbal medicines were produced traditionally would not stand up to legal scrutiny, as many ‘herbal medicines sold over the counter and some practitioner supplies were industrially produced’. Id., p. 30.

56 A. Dixon, *Moving in from the Fringes: The Regulation of Complementary Practitioners in the UK* (2014).

57 Human Medicines Regulations 2012, SI 2012/1916, Regs 3(6) and 3(9).

58 There were several working groups dedicated to the issue of the regulation of herbal medicines and herbalists following the 2000 report on complementary medicine by the House of Lords Select Committee on Science and Technology. House of Lords Select Committee on Science and Technology, *Science and Technology Committee: Sixth Report* (2000), at <https://publications.parliament.uk/pa/ld199900/ldselect/ldsctech/123/12301.htm>.

59 This was reflected in our interviews with representatives of associations for Chinese herbal medicine, Western herbalism, and Ayurveda alike.

60 Diaz Peralta et al., op. cit., n. 25.

61 Hansard, HC, ‘Early Day Motion 279’ (4 December 2002), at <https://edm.parliament.uk/early‐day‐motion/24097/traditional‐herbal‐medicinal‐products‐directive>.

62 For more on these modernization trajectories, see H. Madhavan and J. P. Gaudillière, ‘Reformulation and Appropriation of Traditional Knowledge in Industrial Ayurveda: The Trajectory of Jeevani’ (2020) 14 *East Asian Science, Technology and Society* 603.

63 Interview, representative from an Ayurveda association, 30 May 2018.

64
*Eatweeds*, ‘EP12: Passion Potions’ (2017), at <https://www.eatweeds.co.uk/ep12>.

65
*The Herbarium*, ‘A Network of Transition Herbalists’ *The Herbarium*, 1 February 2009, at <https://theherbarium.wordpress.com/2009/02/01/network‐of‐transition‐herbalists>.

66 Griggs, op. cit., n. 1; P. S. Brown, ‘Herbalists and Medical Botanists in Mid‐Nineteenth‐Century Britain with Special Reference to Bristol’ (1982) 26 *Medical History* 405.

67 Interview, herbalist, 12 July 2018.

68 Interview, herbalist, 5 July 2018.

69 For links to various projects of this kind, see *Grass Roots Remedies*, ‘Links & Library’ *Grass Roots Remedies*, at <https://www.grassrootsremedies.co.uk/links/>.

70 R. Santosh, ‘Practicing Ayurveda in the UK: Simplification, Modification, Hyphenation, and Hybridization’ in *Routledge Handbook of Complementary and Alternative Medicine*, eds N. K. Gale and J. V. McHale (2015) 90.

71 Interview, op. cit., n. 63; *Eatweeds*, op. cit., n. 64.

72 V. Silano et al., ‘Regulations Applicable to Plant Food Supplements and Related Products in the European Union’ (2011) 2 *Food & Function* 710; N. Tejedor‐García et al., ‘The Effect of the European Traditional Use Directive on the Register of Herbal Medicinal Products in Spain’ (2015) 29 *Gaceta Sanitaria* 221.

73 S. R. Craig, *Healing Elements: Efficacy and the Social Ecologies of Tibetan Medicine* (2012) 5.

